# Reflections on Training in Acute Inpatient Psychiatry: Perspectives from Foundation, Core, and Higher Trainees

**DOI:** 10.1192/j.eurpsy.2026.11975

**Published:** 2026-07-31

**Authors:** B. S. Cheema, S. Downes, A. Khan, J. Beezhold

**Affiliations:** 1 Norfolk and Suffolk Foundation Trust, Great Yarmouth; 2 University of East Anglia, Norwich, United Kingdom

## Abstract

**Introduction:**

Psychiatric training involves progressive responsibility and evolving perspectives across career stages. While curricula emphasise competencies (Royal College of Psychiatrists, 2023), fewer studies explore lived experiences of trainees working at different levels within the same setting. Acute inpatient psychiatry, with its clinical intensity and multidisciplinary demands, offers a valuable context for such reflection. Reflective practice is recognised as a tool for professional growth and resilience (Grant et al., 2017). This poster presents reflections from three doctors - a Foundation Year 1 (F1), a Core Trainee Year 1 (CT1), and a Specialist Trainee Year 5 (ST5) - who began work simultaneously on the same acute psychiatric ward.

**Objectives:**

- To explore similarities and differences in experience, responsibility, and learning needs across three training levels.
- To highlight challenges and opportunities in the first month on an acute psychiatric ward.
- To consider how reflective comparison may inform supervision and curriculum development.

**Methods:**

A reflective practice framework was used. Each trainee kept notes during the first month. Themes were discussed and synthesised using narrative thematic analysis. Domains examined included clinical responsibility, supervision, team integration, patient engagement, and emotional impact.

**Results:**

Shared themes included the challenges of managing risk and the steep learning curve of inpatient psychiatry. Distinct perspectives emerged:

- *F1:* Focused on adapting to psychiatric terminology, balancing physical health duties, and gaining confidence in mental state examination.
- *CT1:* Reflected on transitioning from general medicine, taking responsibility for independent assessments, and consolidating diagnostic skills.
- *ST5:* Highlighted leading ward rounds, complex decision-making, and balancing service pressures with teaching juniors.

Shared reflection enhanced individual learning and strengthened team cohesion.

**Conclusions:**

This reflective comparison illustrates how acute psychiatry provides diverse learning experiences tailored to the training stage. Comparing F1, CT1, and ST5 perspectives highlighted complementary challenges and opportunities. Such cross-level reflection can inform supervision strategies, support teamwork, and enrich psychiatric training.

**References:**

1 Grant, A., Townend, M., Mills, J., & Cockx, A. (2017). *Assessment and Case Formulation in Counselling and Psychotherapy.* Sage.

2 Royal College of Psychiatrists. (2023). *Curriculum for Core Psychiatry Training.* London: RCPsych.

**Disclosure of Interest:**

None Declared

